# The IAOx-Dependent IAA Biosynthesis Pathway: Acquired Insights, Paradigm Shifts, and Unresolved Questions

**DOI:** 10.3390/plants15030436

**Published:** 2026-01-30

**Authors:** Ming-Kun Ma, Verena Kriechbaumer, Dong-Wei Di

**Affiliations:** 1 Institute of Agricultural Resources and Environment, Sichuan Academy of Agricultural Sciences, Chengdu 610066, China; mamingkun@scsaas.cn; 2 School of Biological and Medical Sciences, Faculty of Health, Science and Technology, Oxford Brookes University, Oxford OX3 0BP, UK; 3 State Key Laboratory of Soil and Sustainable Agriculture, Institute of Soil Science, Chinese Academy of Sciences, Nanjing 210008, China; 4 University of Chinese Academy of Sciences, Beijing 100049, China

**Keywords:** auxin biosynthesis, indole-3-acetaldoxime, indole-3-acetamide, indole-3-acetonitrile, growth-defense trade-off

## Abstract

The auxin indole-3-acetic acid (IAA) is essential for plant growth and stress adaptation. Its biosynthesis via the indole-3-acetaldoxime (IAOx) pathway has recently undergone a paradigm shift. Recent genetic and metabolomic studies have fundamentally revised the indole-3-acetaldoxime (IAOx) pathway from a linear route (IAOx→IAN→IAM→IAA) to a dynamic network. This review synthesizes this paradigm shift by integrating evidence from key Arabidopsis studies. Crucially, mutants disrupting multiple downstream enzyme families fail to block IAA overproduction in the IAOx-accumulating *superroot 2* (*sur2*) background. Functioning as a central branching point between auxin and defense metabolism, the tryptophan-derived metabolite IAOx, along with indole-3-acetonitrile (IAN) and indole-3-acetamide (IAM), elicits auxin responses via independent, tissue-specific pathways, with no metabolic requirement for IAM as a universal intermediate. Furthermore, IAN and IAM levels do not increase with massive IAOx accumulation, indicating a bypass route from IAOx to IAA. We conclude that IAOx acts as a central metabolic hub, partitioning flux competitively between growth and defense. Resolving the unknown IAOx-converting enzyme, the signaling roles of IAN/IAM, and the logic of metabolic channeling is vital to understanding how plants integrate hormonal and stress responses.

## 1. Introduction

Auxin, a core class of plant hormones, plays a pivotal role in regulating plant development and stress responses [1,2]. It governs diverse physiological processes, including cell division and elongation, tropic movements, apical dominance, flowering, and senescence, while also coordinating adaptations to biotic and abiotic stresses [1,3]. Among the three naturally occurring active auxins, indole-3-acetic acid (IAA), phenylacetic acid (PAA), and 4-chloroindole-3-acetic acid (4-Cl-IAA), IAA stands out as the most ubiquitous and physiologically crucial [4,5]. To ensure normal growth and adaptation in complex environments, plants meticulously regulate IAA levels through a sophisticated homeostatic system. This system relies on a dynamic equilibrium among IAA biosynthesis, polar transport, signal transduction, and the interconversion between its active and inactive forms [6,7,8,9].

IAA biosynthesis serves as the foundation of this regulatory network, originating from chorismate in the chloroplast, which is the terminal product of the shikimate pathway [10]. Biosynthetic routes are primarily classified as tryptophan (Trp)-dependent (TD) or Trp-independent (TI), with both pathways converging at the key metabolic intermediate indole-3-glycerol phosphate (IGP) [10,11]. During Trp synthesis in *Arabidopsis thaliana*, chorismate is converted to anthranilate by the anthranilate synthase (AS). This rate-limiting step is mediated by the *WEI2/ASA1* and *WEI7/ASB1* genes, encoding the α and β subunits of AS enzyme, respectively [12,13,14]. Anthranilate is then sequentially transformed into 1-(O-carboxylphenylamino)-1-deoxyribulose-5-phosphate (CdRP) through the coordinated actions of phosphoribosyltransferase (PAT1) and phosphoribosyl anthranilate isomerase (PAI), ultimately yielding IGP via IGP synthase catalysis [15]. As a critical metabolic branch point, IGP is predominantly channeled to Trp through the tryptophan synthase complex (TSA/TSB) for entry into TD pathways, while also serving as the substrate for indole synthesis via indole synthase (INS) to initiate the TI route [16,17].

Among TD pathways, the indole-3-pyruvic acid (IPyA) pathway represents the predominant and best-characterized route for plant IAA production [2,18]. This pathway involves two core enzymatic reactions: the transamination of L-Trp to IPyA by the TAA1/TAR family of aminotransferases [19,20,21,22], followed by oxidative conversion of IPyA to IAA catalyzed by YUCCA (YUC) family flavin monooxygenases, a reaction recognized as the rate-limiting step [23,24,25,26,27,28]. The identification of the YUC gene family [27], coupled with evidence of its members’ functional redundancy, distinct spatiotemporal expression patterns [18,24,26], and functional coordination with the upstream TAA1/TAR enzymes [23,28], collectively underscores the centrality of the IPyA pathway in plant auxin biosynthesis. The Arabidopsis *YUC* gene family comprises 11 members exhibiting both functional redundancy and spatial specificity, thereby facilitating precise spatiotemporal control of IAA synthesis [18]. Furthermore, this pathway is further modulated by feedback regulation through the aminotransferase REVERSAL OF SAV3 PHENOTYPE 1 (VAS1), which catalyzes IPyA reconversion to Trp to maintain metabolic homeostasis [29].

Beyond the IPyA pathway, multiple additional Trp-derived routes contribute to IAA biosynthesis. The indole-3-acetamide (IAM) pathway involves IAM hydrolysis to IAA by amidase 1 (AMI1), its homologs (such as TOC64s and FAAHs), or the recently identified IAM hydrolases (IAMH1/2), though the endogenous origins of IAM remain partially elusive, potentially deriving from IAOx conversion or unidentified sources [30,31]. Although the tryptamine (TAM) pathway has been proposed, its physiological significance remains controversial, with potential functionality restricted to specific tissues or particular conditions [32]. TAM exhibits auxin-like activity, but several lines of evidence challenge its role as a major IAA precursor: it is often found at high, non-specific levels across species, originates from distinct Trp pools, and its proposed conversion to IAA lacks robust enzymatic support [33,34,35]. While tissue-specific conversion (e.g., in pea roots) suggests limited, context-dependent functionality, TAM is not considered a conserved, primary route for IAA biosynthesis [2]. In contrast, the indole-3-acetaldoxime (IAOx) pathway, which is especially prominent in Brassicaceae species, constitutes a major TD branch that is intricately linked to specialized metabolism and stress responses, and will be the primary focus of this review [26,27].

The TI pathway, which bypasses Trp, is postulated to initiate from IGP through direct conversion to indole via INS, followed by poorly defined metabolic steps ultimately yielding IAA [16,17,35]. These studies, using genetic and isotopic tracing evidence, suggested a potential role for this pathway in embryogenesis [17] and maintaining basal IAA levels [16], although its precise enzymatic mechanisms remain elusive to date [35]. This pathway is hypothesized to function during embryogenesis and in maintaining basal IAA levels, though its enzymatic mechanisms and regulatory networks remain substantial challenges for future investigation [16,17]. Crucially, the entire IAA biosynthetic network operates under multi-layered regulation encompassing epigenetic modifications, transcriptional control, post-transcriptional processing, post-translational modifications, and metabolic feedback, all functioning coordinately to maintain IAA dynamic balance and support plant growth and environmental adaptation [6,18,36]. This involves integrated control at multiple levels, from epigenetic and transcriptional regulation [6,36] to the precise spatiotemporal control of key biosynthetic genes like *YUC* [18].

The field of auxin biology has undergone a profound paradigm shift, decisively moving beyond the confines of the canonical nuclear Skp1-Cullin1-F-box (SCF) TIR1/AFBs- Auxin/Indole-3-Acetic Acid (Aux/IAA)- Auxin Response Factor (ARF) signaling pathway to embrace a more complex, decentralized, and multi-layered signaling network. This transformation is underscored by the establishment of a sophisticated dual-stream mechanism: one consisting of a rapid, cell-surface signaling cascade mediated by the Auxin-Binding Protein (ABP1)/ABP1-Like protein (ABL)-Transmembrane Kinase (TMK) module, which directly phosphorylates key targets like PIN-FORMED (PIN) efflux carriers and plasma membrane H^+^-ATPases to coordinately regulate auxin transport and the acid growth process within minutes [37,38,39,40,41,42,43]; and a refined view of nuclear signaling, in which the TIR1/AFB receptors depend on their intrinsic adenylate cyclase activity to generate local cAMP pools essential for full transcriptional reprogramming of auxin-responsive genes [8,44,45,46]. This bifurcation elegantly resolves long-standing questions about the temporal disconnect between rapid physiological responses and slower transcriptional changes [8,37,38].

Building on this conceptual framework, recent advances have further elucidated the self-organizing properties of the auxin system. Studies on self-organizing transport modules, together with high-resolution structural insights into AUX1/LAX influx and PIN efflux carriers, have revealed fundamental biophysical principles governing the spatiotemporal control of auxin distribution [38,47,48,49,50]. These findings bridge signaling and transport, illustrating how auxin can rapidly modulate its own directional movement through non-transcriptional pathways while also generating complex distribution patterns that feed back into both signaling branches. Through intricate feedback loops and crosstalk with other hormonal and environmental signals, this integrated network provides a robust molecular basis for developmental plasticity, from root gravitropism to phyllotaxis.

Despite these advances, significant knowledge gaps persist between the well-characterized IPyA pathway and less understood routes such as TAM, IAM, and particularly IAOx [2,51]. The IAOx pathway has recently undergone significant conceptual revisions, with transformative insights from seminal work by Fenech et al. (2025) compelling a comprehensive re-evaluation of its metabolic fluxes and physiological roles [52]. Against this backdrop of renewed interest and evolving paradigms, this review systematically traces the research trajectory of the IAOx pathway, providing an overview of established conceptual frameworks, a critical analysis of emerging evidence, and an identification of key scientific questions for future investigation. It should be noted that the mechanistic insights and paradigm shifts discussed herein derive primarily from studies in Brassicaceae species, notably Arabidopsis.

## 2. The Established Paradigm: IAOx as the Central Precursor and Linear Pathway

**Figure 1:**
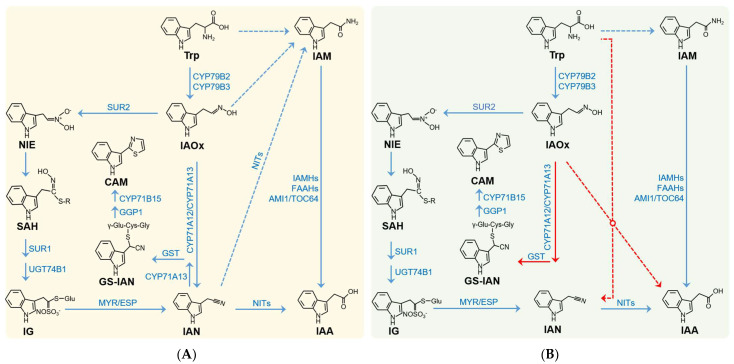
Paradigm shift in the IAOx-dependent auxin biosynthesis pathway: An evolving view. (**A**) The established paradigm: A linear IAOx-dependent auxin biosynthesis pathway. (**B**) An emerging view: IAOx-dependent auxin biosynthesis as a complex metabolic network. Abbreviations: CYP79B2/B3, Cytochrome P450 Family 79 Subfamily B Polypeptide 2/3; SUR1, SUPERROOT 1; SUR2, SUPERROOT 2 (CYP83B1); UGT74B1, UDP-Glycosyltransferase 74B1; MYR, Myrosinase; ESP, Epithiospecifier Protein; CYP71B15, Cytochrome P450 Family 71 Subfamily B Polypeptide 15 (PAD3); GGP1, General Regulator of Peptidase 1; GST: Glutathione S-Transferase; CYP71A12/13, Cytochrome P450 Family 71 Subfamily A Polypeptide 12/13; NIT, Nitrilase; IAMH, Indole-3-Acetamide Hydrolase; FAAH, Fatty Acid Amide Hydrolase; AMI1, Amidase 1; TOC64: Translocon of the Outer Membrane of the Chloroplasts 64; Trp, Tryptophan; IAOx, Indole-3-Acetaldoxime; NIE, 1-aci-Nitro-2-Indolyl-Ethane; SAH, S-alkyl-thiohydroximate; IAN, Indole-3-Acetonitrile; GS-IAN, Glutathione Conjugate of Indole-3-Acetonitrile; IAM, Indole-3-Acetamide; IG, Indole Glucosinolate; CAM, Camalexin; IAA, Indole-3-Acetic Acid. All depicted biochemical pathways and molecular structures were drawn based on established knowledge from the cited literature.

The IAOx pathway represents a classic model for the biosynthesis of the plant auxin IAA, primarily characterized in Brassicaceae species. This model, systematically articulated by Sugawara et al. [34], posits a linear metabolic cascade: IAOx is converted to IAN by cytochrome P450 enzymes of the CYP71A family (e.g., CYP71A12/A13) [53,54,55]. IAN is subsequently transformed into IAA and/or IAM by nitrilases (NITs) [51,56,57,58,59,60]. Finally, the conversion of IAM to IAA is mediated by a range of enzymes, encompassing amidase AMI1, its homologs (e.g., TOC64s and FAAHs), as well as the recently characterized IAM hydrolases IAMH1 and IAMH2 [30,61,62,63,64,65] (Figure 1A). Strong genetic support for this model comes from studies of the *sur2* mutant. By blocking the diversion of IAOx into indole glucosinolate (IG) biosynthesis, *sur2* leads to substantial IAOx accumulation, and this accumulated IAOx is then channeled into IAA production, resulting in characteristic high-auxin phenotypes such as shortened roots, disintegrated hypocotyls, and epinastic cotyledons [66,67].

Early investigations detected IAOx unequivocally in a few Brassicaceae species, such as Arabidopsis, while it was generally absent in non-Brassicaceae species such as tomato, rice, maize, pea, and tobacco, suggesting potential species specificity [15,34,68]. However, the synthesis and metabolic flux of IAOx are significantly enhanced under various stress conditions, including pathogen infection (e.g., *Piriformospora indica*) [69], high temperature [70], or salt stress [71], indicating its crucial physiological role in plant stress responses. Building on this view, subsequent studies have reported significant IAOx accumulation in specific contexts beyond Brassicaceae, such as in maize following herbivory [72,73] and in *Medicago truncatula* under iron deficiency [74]. These observations demonstrate that IAOx metabolism can be activated by specific environmental cues beyond the Brassicaceae family. A key question emerging from this finding is whether the associated auxin phenotypes in non-Brassicaceae species rely on CYP79 homologs or operate through entirely distinct mechanisms. Furthermore, exogenous IAOx application in *Medicago truncatula* induces typical auxin responses reminiscent of the Arabidopsis glucosinolate-deficient mutants *sur1* and *sur2*, including increased lateral root formation and suppressed primary root elongation [75,76]. These observations demonstrate that IAOx metabolism or perception can be activated in non-Brassicaceae species, yet the enzymatic basis for IAOx conversion to IAA in these species remains unclear and may not involve orthologs of the Arabidopsis CYP79B or the unknown enzyme implied by the *sur2* studies.

In Arabidopsis, the biosynthesis of IAOx, catalyzed by CYP79B2/B3, constitutes the rate-limiting step for its downstream metabolism [70,76,77] (Figure 1). Overexpression of CYP79B2 leads to IAOx accumulation and consequent IAA overproduction phenotypes, such as elongated hypocotyls, leaf epinasty, and elevated IAA levels. Conversely, the *cyp79b2/b3* double mutant lacks IAOx and exhibits reduced IAA levels and growth defects under high-temperature stress [34,70]. Beyond being a mere precursor of IAA, IAOx acts as a critical metabolic hub [78]. Its flux is precisely directed toward IG biosynthesis by CYP83B1/SUR2 [66,67,79], or, under inducing conditions, channeled into IAN by CYP71A12/CYP71A13 [53,55] (Figure 1). It is noteworthy that IG can be converted to IAN through catalysis by MYR and ESP [31,80,81]. IAN is subsequently conjugated to form GS-IAN, which is then utilized by GGP1 and CYP71B15 for the synthesis of the phytoalexin camalexin (CAM) [52,78].

The traditional model is underpinned by multiple lines of evidence consolidating IAM and IAN as key intermediates [82]. Genetically, the *cyp79b2/b3* double mutant shows significantly reduced levels of IAM and IAN, whereas glucosinolate-deficient mutants *sur1/sur2*, which accumulate IAOx, exhibit elevated IAM and/or IAN levels, driving IAA overproduction [34,76,83]. Biochemically, isotope tracing experiments demonstrated the conversion of exogenous IAOx to IAM and IAN [34], and CYP71A12/CYP71A13 have been confirmed to convert IAOx to IAN *in vitro* and *in vivo* [55]. The nitrilases NIT1-3 and the amidase hydrolases AMI1/IAMH have been identified as key enzymes catalyzing the hydrolysis of IAN and IAM to IAA, respectively [30,51,56,57,58]. Furthermore, cross-species studies reinforce this connection. In IAOx-treated Medicago plants, significant IAN accumulation was detected in shoots and in roots of iron-deficient plants, indicating that IAOx can be converted to IAN in roots or after translocation to shoots, a process occurring under both iron-deficient and sufficient conditions [74].

Nevertheless, as of 2025, several fundamental questions remain unresolved. First, the complete linear conversion route from IAOx to IAA is not fully established. While CYP71A13 catalyzes IAOx→IAN, the enzyme(s) responsible for the direct conversion of IAOx to IAM or other potential intermediates remain unknown [83]. Second, the necessity of IAN and IAM under basal growth conditions is questionable, as higher-order *nit* and *ami1 iamh* mutants do not exhibit significant IAA-deficient phenotypes under normal growth. This suggests functional redundancy or a primary role for these pathways outside of maintaining basal growth [30,51,56,83]. Furthermore, a conceptual gap persists between the apparent restriction of the IAOx pathway to Brassicaceae and its broader importance in stress responses [34,68]. Finally, the regulatory mechanisms governing the ‘channeling’ of metabolites between different pathways, such as the potential physical or metabolic separation of the IAN pool dedicated to CAM biosynthesis from the pool potentially involved in IAA production, remain unclear [54,84]. These outstanding issues collectively define the critical research frontiers in the field beyond 2025.

## 3. Contemporary Revisions: Redefining the Pathway Through Network Regulation

Prior to the study by Fenech et al. (2025) [52], research on IAOx-mediated IAA biosynthesis was governed by a classic theoretical framework depicting a linear metabolic pathway (Figure 1A). However, Fenech et al. [52] fundamentally challenged this model through systematic genetic and metabolomic analyses (Figure 1B). Through a comprehensive genetic approach involving the generation and analysis of higher-order mutants targeting entire gene families (*cyp71a12 a13 a18*, *nit1 nit2 nit3 nit4*, *ami1 toc64-III toc64-V faah1 faah2*), combined with detailed metabolomic profiling, Fenech et al. (2025) provided compelling evidence that challenges the established linear model [52]. Notably, none of these mutants exhibited developmental abnormalities under standard conditions, in contrast to the weak auxin-deficient mutant *wei8/sav3/ckrc1* [19,20,21]. This absence of phenotypic defects clearly indicated that these gene families are dispensable for basal auxin biosynthesis.

A pivotal breakthrough came from systematically introducing these higher-order mutations into the *sur2* background, where the IAOx pathway is hyperactive. The linear model predicted that disrupting any downstream step in this background should suppress the high-auxin phenotypes by blocking IAOx conversion to IAA (Figure 1A). Contrary to this prediction, and inconsistent with the linear model, all mutant combinations fully retained the characteristic *sur2* phenotypes at both seedling and adult stages [52]. In stark contrast, the *cyp79b2 cyp79b3 sur2* triple mutant, which blocks IAOx production at the source, completely suppressed these phenotypes. This collective genetic evidence strongly demonstrates that the *CYP71As*, *NITs*, and *AMI/TOC64/FAAHs* gene families are not required for the excessive IAA production in *sur2* [52].

Further pharmacological and reporter gene analyses demonstrated that IAOx, IAN, and IAM engage in intricate tissue-specific metabolic interactions. Although all three compounds activated *p*DR5:GFP expression and induced auxin-like physiological responses, their effects were organ-dependent. IAOx exerted its primary influence in root tissues, IAM specifically enhanced hypocotyl elongation, and IAN was active in both roots and hypocotyls [52]. To decipher whether these distinct phenotypes reflected interconnected or parallel pathways, the researchers leveraged their mutant collection. Crucially, the *nit1 nit2 nit3 nit4* mutant was root-insensitive to IAN but responded normally to IAOx. Similarly, the *iamh1 iamh2* mutant was hypocotyl-insensitive to IAM but responded normally to IAOx and IAN. These results definitively show that the conversion of IAOx and IAN to IAA does not require IAM as an intermediate (Figure 1). Furthermore, the normal IAM response in higher-order AMI family mutants suggests that IAM-to-IAA conversion is primarily mediated by IAMHs, not the AMI family [30].

Metabolomics data provided direct quantitative evidence contradicting the linear model. In *sur2* and its higher-order mutants, despite a dramatic 60- to 120-fold accumulation of IAOx, the putative downstream intermediates IAN and IAM failed to accumulate and were even reduced. A decisive finding came from the *ami1-2 toc64-III toc64-V faah1 faah2 sur2* and *iamh1 iamh2 sur2* mutants, where IAM accumulation was anticipated if it were a central intermediate. The absence of significant IAM accumulation in these lines is entirely inconsistent with the linear model and strongly supports the existence of an unknown route from IAOx to IAA that bypasses both IAN and IAM.

In summary, the work by Fenech et al. presents a compelling rebuttal of the linear IAOx pathway model and establishes a robust, evidence-based framework for understanding IAOx metabolism in Arabidopsis [52]. This framework is founded on three key pillars supported by integrated genetic, pharmacological and metabolomic data: (1) the existence of an efficient alternative pathway from IAOx to IAA that is hyperactivated in the *sur2* mutant; (2) the operational independence of this pathway from the *CYP71A*, *NIT*, *AMI/TOC64/FAAH* and *IAMH* gene families; and (3) the parallel and metabolically decoupled roles of IAOx, IAN and IAM as precursors, each engaging tissue-specific routes to produce IAA via distinct primary mechanisms, an unknown enzyme for IAOx, NITs for IAN and IAMHs for IAM [30,51,55,56,85]. This evidence-based model conclusively defines what is known, the existence and independence of this pathway, while starkly revealing what remains unknown: the biochemical identity of the conversion mechanism itself.

## 4. Future Research Perspectives: Unresolved Mysteries and Future Frontiers

The study by Fenech et al. has overturned the traditional linear model of IAOx-mediated IAA biosynthesis, representing a paradigm shift in the field (Figure 1B). Rather than concluding the research journey, this work reveals a more complex and highly networked regulatory landscape, and, most importantly, defines the precise boundaries of our current ignorance. The following directions constitute the most critical research frontiers within this revised framework. Crucially, the discussions below on mechanisms and origins are informed speculations derived from established evidence, not extensions of the evidence itself.

(i) The central enigma: The unidentified enzyme(s) catalyzing IAOx-to-IAA conversion and its regulation

The most pressing unresolved question, revealed by the work of Fenech et al. (2025) [52], is the molecular identity of the enzyme(s) responsible for the efficient conversion of accumulated IAOx to IAA in the *sur2* mutant. While its existence is robustly inferred from the inability of mutation in *CYP71A*, *NIT*, and *AMI* families to suppress *sur2* phenotypes, its molecular identity, biochemical properties, and regulatory mechanisms remain entirely unknown. While the genetic and metabolomic evidence unequivocally demands the existence of an efficient IAOx-to-IAA conversion route, its biochemical mechanism remains unresolved. Several plausible hypotheses merit consideration: a single unidentified enzyme (e.g., a novel oxidoreductase, lyase or dioxygenase) could catalyze direct conversion; a multi-enzyme metabolon, akin to the camalexin biosynthetic complex, might channel IAOx through transient association of catalytic and auxiliary subunits; strict subcellular compartmentalization (e.g., in peroxisomes or specialized ER domains) could provide both the requisite catalytic milieu and regulatory isolation; or an enzyme-triggered chemical step might generate a reactive intermediate that rapidly rearranges to IAA in the cellular context. These speculative models are grounded in the logical imperative of the genetic data and in known biochemical principles, and their experimental dissection represents the immediate biochemical frontier in the field.

Future research must focus on identifying its encoding gene(s), employing strategies such as genetic suppressor screens, genome-wide association studies, or interactome proteomics. Subsequently, profound regulatory questions emerge: Is the activity of this enzyme finely regulated by upstream signaling pathways? Does a competitive or mutually exclusive regulatory relationship exist between this enzyme and defense-related enzymes (e.g., CYP71A12/13) for the common substrate IAOx? Particularly under stress conditions such as pathogen infection, is this enzyme directly suppressed by core defense transcription factors like WRKY33 [69], thereby channeling metabolic flux preferentially toward defense pathways? Elucidating these mechanisms will not only identify a key missing component in auxin metabolism but also reveal a critical control point in the plant’s stress response network.

(ii) Re-evaluating the physiological origins and functions of IAN and IAM

With IAN and IAM being excluded from the primary route of IAOx-to-IAA conversion, their physiological roles demand a critical re-examination that goes beyond their former status as pathway intermediates. Emerging evidence suggests that IAN and IAM not only contribute to systemic IAA biosynthesis but also exhibit distinct tissue-specific signaling functions in planta. IAM accumulation and AMI1 expression are particularly pronounced in reproductive and juvenile tissues such as flowers, young leaves, and developing seeds, indicating a potential role in localized auxin production during reproductive development and embryogenesis. In contrast, IAN is enriched in root tips, lateral root primordia, and leaf margins, suggesting a spatially restricted role in organogenesis and stress-responsive growth regulation. These observations highlight the nuanced, tissue-partitioned contributions of IAN and IAM to auxin-mediated signaling, beyond their canonical roles as mere biosynthetic intermediates.

The immediate task is to elucidate their endogenous biosynthetic pathways in wild-type plants. For instance, does IAM originate from a yet-unidentified, independent pathway involving the direct amidation of tryptophan? More significantly, IAN and IAM may function not merely as auxin precursors but as output signals of distinct metabolic decisions. IAN, derived from glucosinolate hydrolysis, for example, could act as a ‘danger signal’ that triggers specific defense responses locally or systemically. This possibility raises several pivotal questions: Does the plant cell possess sophisticated sensing mechanisms, such as NLR-like immune receptors or specific kinases, capable of discriminating IAN from different origins? Furthermore, how does the cell prevent IAN generated from defense pathways from inappropriately activating auxin signaling cascades? However, several key experimental gaps persist in fully elucidating these signaling roles. First, tissue and cell-type-specific functions remain unclear due to the lack of high-resolution localization studies and tissue-specific genetic manipulations. Second, the mechanisms by which IAN/IAM signaling integrates with canonical auxin perception pathways (e.g., TIR1/AFB-Aux/IAA-ARF) require systematic genetic and biochemical validation. Third, the environmental modulation of these pathways, particularly under drought, pathogen challenge, or nutrient stress, remains poorly mapped at the molecular level. Finally, the evolutionary conservation and functional divergence of IAN/IAM signaling across plant species warrant comprehensive comparative studies.

These questions position IAN and IAM as central players in an ‘IAOx metabolic decision-making system’. Delving into these mechanisms is, therefore, essential to understand how this system governs the core life strategy of plant growth-defense trade-offs, a theme that is intrinsically linked to metabolic channeling and environmental signal integration. To address these gaps, future research should prioritize: (1) deploying CRISPR/Cas9-based tissue-specific editing and single-cell metabolomic imaging to resolve spatial dynamics of IAN/IAM accumulation and conversion; (2) conducting interactome screenings and genetic epistasis analyses to identify signaling components that interface with IAN/IAM metabolic nodes; (3) establishing multifactorial stress regimes to dissect environment-dependent regulation of IAN/IAM pathways; and (4) expanding phylogenetic-functional analyses across monocot and eudicot lineages to trace the evolution of IAN/IAM-mediated signaling modules.

(iii) The regulatory logic of metabolic channeling and the determinative role of cell identity

Substantial evidence indicates that IAOx metabolism exhibits remarkable ‘channeling’ or ‘compartmentalization’, whereby the identical precursor is precisely directed into distinct downstream pathways. For instance, pathogen infection can almost completely shut off the flux of IAOx into IG while simultaneously enhancing its conversion to the phytoalexin CAM. The molecular mechanisms underlying this precise metabolic reprogramming, particularly its upstream regulatory logic, remain elusive. A central hypothesis posits that this regulation may rely on cell type-specific ‘preset metabolic programs’. In other words, particular cell types, due to their inherent transcriptomic signatures, their ‘cell identity’, may be intrinsically biased toward channeling IAOx into a specific metabolic route. Critically, metabolic channeling is not a static configuration but is dynamically reshaped by developmental and environmental cues. For example, under pathogen attack, transcriptional reprogramming mediated by WRKY33 may suppress the unknown IAOx-to-IAA converting enzyme while upregulating *CYP71A12/13*, thereby redirecting IAOx flux toward camalexin synthesis. Such dynamic repartitioning of metabolic flux likely involves rapid post-translational modifications, allosteric regulation, or the reversible assembly of enzyme complexes. Furthermore, this channeling is likely underpinned by precise subcellular organization and protein–protein interactions. The biosynthesis of camalexin, for instance, involves a metabolon comprising CYP71A12, CYP71A13, GGP1, and GSTU4, which spatially coordinates intermediate transfer and prevents diffusion of reactive metabolites. By analogy, the unknown route from IAOx to IAA may operate within dedicated subcellular niches, such as specialized endoplasmic reticulum domains or peroxisomes, or through transient enzyme complexes that physically segregate auxin production from defense-related pathways. Future research must leverage cutting-edge technologies such as single-cell metabolomics and spatial transcriptomics to construct a high-resolution spatiotemporal atlas of the IAOx metabolic network in planta. Integrating these approaches will be essential to map context-dependent channeling events and elucidate how plants toggle between metabolic programs in real time. This approach will allow us to move beyond tissue-level averaging and ultimately uncover the cellular basis and fundamental principles driving metabolic path decisions. Understanding this regulatory layer may also provide insights into the evolution of species-specific metabolic strategies.

(iv) Evolutionary trajectory and the mechanisms underlying species specificity

The IAOx pathway, as currently understood from genetic dissection in Arabidopsis, exhibits remarkable complexity within Brassicaceae, yet the evolutionary drivers and molecular basis for this remain incompletely understood. Does this specificity primarily stem from innovations in Brassicaceae-specific cis-regulatory elements, or is it driven by the adaptive evolution of key enzymes such as CYP79B? Conversely, in non-Brassicaceae species like maize and *Medicago truncatula*, are the enzymatic bases for the auxin responses triggered by IAOx or its analogs homologous to the newly discovered unknown IAA biosynthesis pathway in Arabidopsis? A key goal is to determine whether the unknown enzyme in Arabidopsis has functional analogs in other species, and how cell-specific regulatory logics have evolved to shape pathway diversity.

(v) Network integration and signal crosstalk under environmental stress

Within the new metabolic network framework, a central question emerges: how do diverse environmental stresses, such as high temperature, salinity, and pathogen attack, orchestrate resource allocation within the IAOx pathway? Specifically, how are stress signals perceived and transduced to prioritize the flux of IAOx toward defense metabolites like CAM, while simultaneously fine-tuning local IAA levels to coordinate adaptive growth responses? Deciphering the mechanisms governing resource partitioning and signal crosstalk between auxin biosynthesis and defense metabolism under stress conditions holds significant theoretical importance. In this context, the allocation of IAOx flux provides a mechanistic basis for classical growth–defense trade-offs. Rather than viewing IAA and defense metabolites as endpoints of a single linear pathway, the revised model posits IAOx as a central resource node whose flux is partitioned through competing, and often mutually exclusive, enzymatic commitments (Figure 1B). Key determinants of this partitioning include (1) the relative activity and substrate affinity of the unknown IAOx-to-IAA enzyme versus CYP71A12/13 (for CAM) and SUR2 (for IG); (2) their spatiotemporal expression and subcellular localization; and (3) post-translational regulation that rapidly toggles these activities in response to signals. For example, pathogen-triggered suppression of the IAOx-converting enzyme (potentially via WRKY33) coupled with induction of *CYP71A12/13* would establish a hardwired metabolic switch that prioritizes defense (CAM) at the expense of local auxin biosynthesis. Conversely, developmental cues favoring growth may enhance the expression or activity of the unknown enzyme, thereby channeling IAOx toward IAA production.

This network-based view fundamentally revises traditional interpretations of hormone–defense crosstalk. The former linear model implied a direct metabolic coupling in which defense intermediates (e.g., IAN derived from IG hydrolysis or CYP71A13 activity) served as obligatory precursors for auxin, creating a paradox whereby defense activation could inadvertently stimulate growth. The new model decouples these pathways: IAN and IAM are not required intermediates for IAOx-derived IAA. Instead, they arise from parallel, independently regulated branches (e.g., IG hydrolysis for IAN, or yet-unidentified routes for IAM) and can themselves function as tissue-specific auxin precursors or signaling molecules. Thus, crosstalk is not an obligatory metabolic linkage but a competitive, regulatory relationship centered on the IAOx branch point. Hormone and defense outputs can be coordinated or antagonized through transcriptional and allosteric control of the enzymes that compete for IAOx, enabling a more flexible and context-dependent balance between growth and defense. Furthermore, it unveils potential targets for future crop improvement strategies aimed at precisely manipulating the growth-defense balance.

## 5. Conclusions

The IAOx-dependent IAA biosynthesis pathway, once viewed as a linear metabolic route, has now emerged as a dynamic, channeled network that bridges auxin production with defense metabolism. The paradigm shift initiated by recent genetic and metabolomic studies not only redefines the role of IAOx, IAN, and IAM but also reveals a sophisticated regulatory logic governing resource allocation between growth and defense programs.

Looking forward, several frontier challenges remain. Future research must prioritize the identification of the unknown enzyme(s) catalyzing IAOx-to-IAA conversion, elucidate the tissue-specific signaling functions of IAN and IAM, and unravel the mechanisms of metabolic channeling under environmental stress. Moreover, integrating the IAOx pathway into the broader auxin signaling network, encompassing both nuclear and cell-surface signaling modules, will be essential to understand how plants dynamically balance growth and defense in fluctuating environments. Advancing these questions will require interdisciplinary approaches, including single-cell omics, spatial metabolomics, and systems biology modeling, to move from a descriptive framework toward a predictive understanding of auxin-driven plant adaptation.

## Data Availability

Data are contained within the article.
